# Remote engineering of particle-like topologies to visualise entanglement dynamics

**DOI:** 10.1038/s41377-026-02443-x

**Published:** 2026-09-02

**Authors:** Fazilah Nothlawala, Bereneice Sephton, Pedro Ornelas, Mwezi Koni, Bruno Piccirillo, Liang Feng, Isaac Nape, Vincenzo D’Ambrosio, Andrew Forbes

**Affiliations:** 1 School of Physics, University of the Witwatersrand, Private Bag 3, Wits, 2050 South Africa; 2 Dipartimento di Fisica, Università di Napoli Federico II, Complesso Universitario di Monte S. Angelo, Via Cintia, Napoli, 80126 Italy; 3 Department of Electrical and Systems Engineering, University of Pennsylvania, Philadelphia, 19104 PA USA

**Keywords:** Quantum optics, Quantum optics

## Abstract

Skyrmions are a particle-like topology with a quantized skyrmion number, realized across condensed matter and photonic platforms alike. In quantum photonics, they constitute an emerging resource, promising robust quantum information encoding, so far realized as single-photon and bi-photon entangled states. Here we report the first visualization of tripartite entanglement dynamics through topological structure using spin-skyrmion entangled states, where the topology of a single photon is remotely controlled through the spin of its entangled partner. We visualize our tripartite state theoretically by introducing the notion of a topological Bloch sphere that completely captures the entanglement and topological features of the state. By leveraging this state, we realize the first quantum multiskyrmions, comprising multiple localized skyrmions within a single structure, that emulate signatures of their magnetic counterparts. We verify this experimentally and show that traversing our topological sphere reveals entanglement-driven particle-like motion of the localized topological structures. These dynamics unveil a physical manifestation of tripartite entanglement correlations which we illustrate by example of GHZ-like states, enabling a visualization of multiple Bell states encoded within our system. Our work opens exciting possibilities for quantum sensing by mapping complex quantum channel features onto topological observables of multipartite states and offers a promising avenue for harnessing quantum topologies for multi-level encoding quantum communication schemes.

## Abstract (web summary)

Measuring one photon’s spin remotely engineers topology in its entangled partner, creating quantum multiskyrmions with particle-like motion that reveal entanglement dynamics and enable robust quantum sensing and communication.

## Graphical abstract

**Figure:**
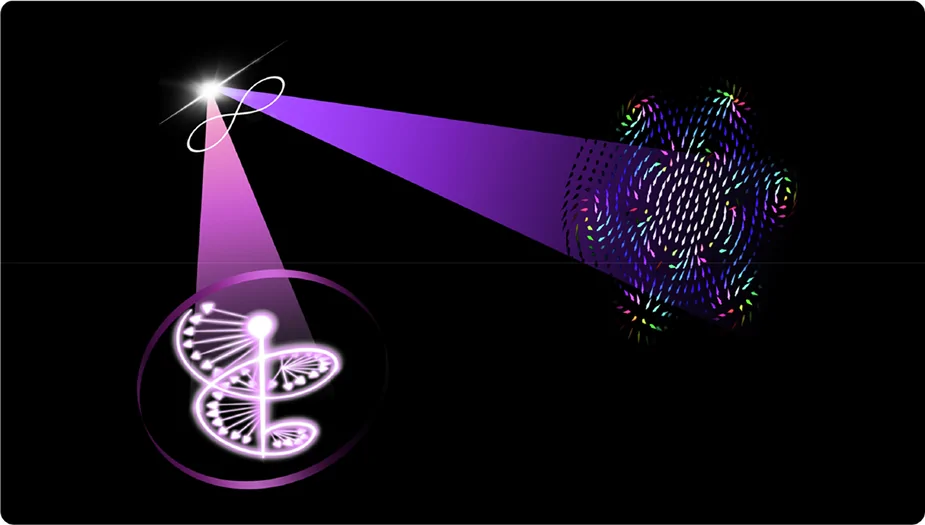

## Introduction

Originally introduced as solitonic solutions in nonlinear field theories^1^, skyrmions are a particle-like topology characterized by an integer-valued topological charge, known as the skyrmion number (*n*), which is associated with the wrapping of a spin or pseudospin field. These topological excitations have since been realized in many systems, from magnetic materials^2,3^, ultracold atomic gases^4,5^, twistronics^6^, liquid-crystals^7,8^ and acoustics^9,10^ to optical fields^11–13^. Underpinned by topological stability, they form attractive candidates for the storage and transfer of information^14–16^.

Their optical implementation has been explored with surface plasmon polaritons^17–19^, evanescent fields^20^, spatiotemporal pulses^21,22^, Poynting vectors^23^ and Stokes parameters of structured paraxial beams^24–26^. In the Stokes approach, the spatial phase and polarization are engineered to produce these spin-textured fields in free space, with the Stokes skyrmion defined as a mapping between the tranverse spatial plane, ${{\mathcal{R}}}^{2}$ and a polarization state space, ${{\mathcal{S}}}^{2}$, represented by the Poincare sphere. These optical Stokes skyrmions offer the advantage of a versatile platform for investigating exotic topologies^26^, light-matter exchange^27–29^ for future data storage devices^30,31^ and the promise of computing^32^ as well as optical processing at the speed of light.

Only recently have these structures been realized in the quantum regime, as non-local states across entangled photons^33^, locally in single photons^34^ and controllably in both regimes (local and non-local)^35^. The topology in these systems, however, has remained a well-defined feature of the underlying states from which they are derived.

Here, we introduce spin-skyrmion entangled states, where the skyrmion topology of one photon is remotely controlled by its entangled twin, with the underlying topological structure revealing tripartite entanglement dynamics. We demonstrate these features through experimentally measured dual-wavelength entangled states, which exhibit topological transitions, switching between two different skyrmion numbers. To visualize this skyrmion number transition, we introduce the notion of a topological Bloch sphere, capturing the state of the skyrmion-photon given measurements on the spin-photon. Notably, no well-defined spin texture or skyrmion number exists prior to measurement of the entangled spin-photon, underscoring the non-local character of the topology. This allows us to report the first creation of quantum multiskyrmions—complex skyrmion structures characterized by multiple skyrmion numbers—exhibiting remotely driven quasiparticle dynamics, emulating magnetic systems. Lastly, we isolate embedded GHZ-like states whose behavior can be identified with the evolution of their topological spin-textures, thereby revealing a physical manifestation of complex tripartite entanglement dynamics. These results open new avenues for harnessing dynamic topological quasiparticles within quantum sensing protocols by encoding complex channel features onto topological observables, enabling physical observation of multipartite state evolution.

## Results

### Non-local topological control

**Fig. 1:**
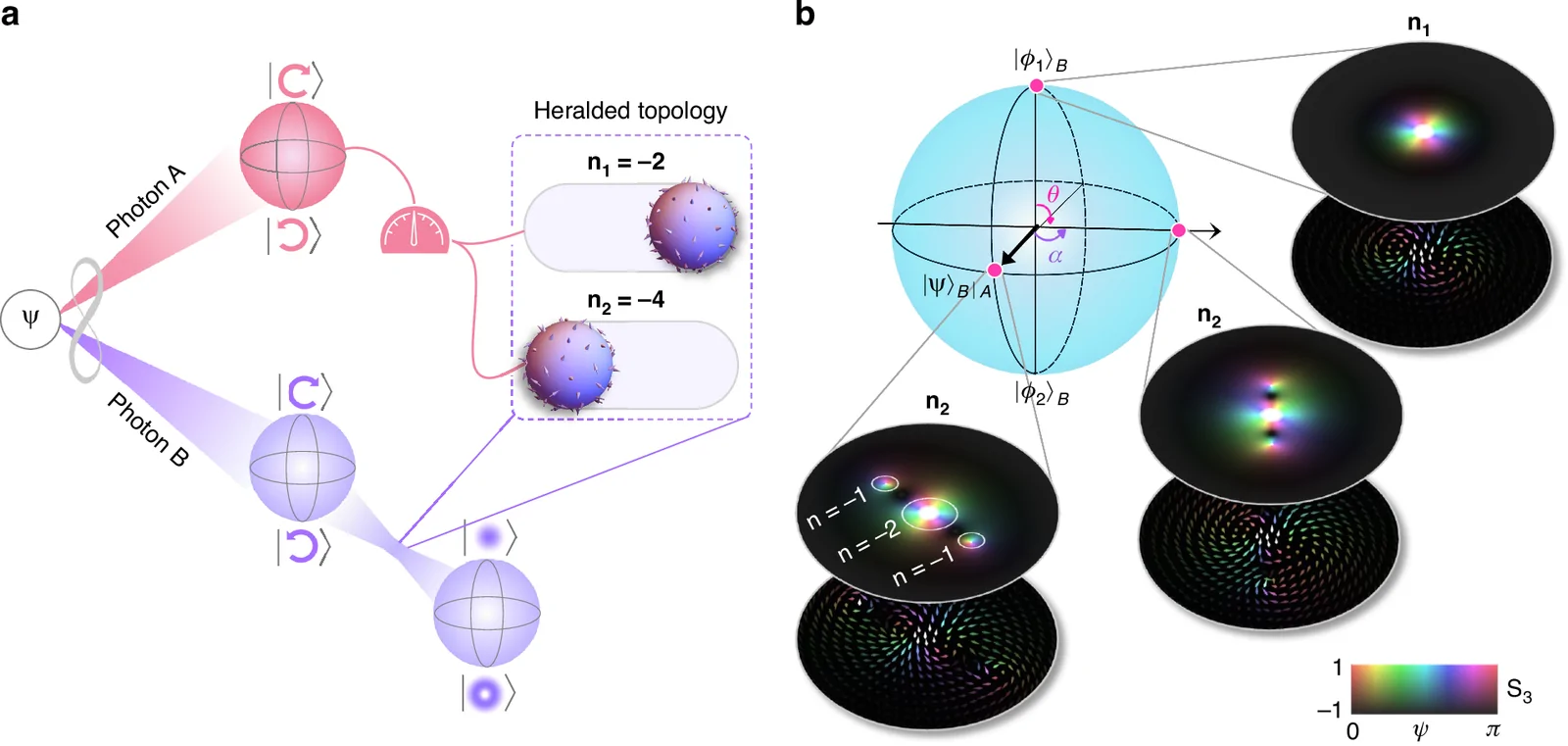
Non-local topological control and creation of quantum multiskyrmions. **a** Photons A and B form an entangled state, where the polarization of photon A is coupled to a skyrmion state on photon B. A measurement made on the polarization of photon A will collapse the state of photon B into a certain skyrmion state with a topological skyrmion number that can be switched between *n*_1_ and *n*_2_. **b** Varying the polarization measurement on photon A allows us to traverse the topological landscape of the spin-skyrmion entangled state, revealing a quantum multiskyrmion composed of quasiparticle-like distributions at the equator of the sphere, and a higher order skyrmion at the poles (represented in the polarization vector plots where $\psi =\frac{1}{2}{\tan }^{-1}({S}_{2}/{S}_{1})$). The emergence of the different topological structures gives rise to a topological switch, yielding a skyrmion number of *n*_1_ at the poles of the sphere, and *n*_2_ at the equator

To enable heralded non-local control of local topology, we begin with the quantum state

1 $${\left\vert \Psi \right\rangle }_{AB}={\left\vert R\right\rangle }_{A}{\left\vert {\phi }_{1}\right\rangle }_{B}+{\left\vert L\right\rangle }_{A}{\left\vert {\phi }_{2}\right\rangle }_{B}$$

where the polarization of photon A (denoted by orthogonal right- and left-circularly polarization states, $\left\vert R\right\rangle ,\left\vert L\right\rangle$) is entangled with skyrmion states $\left\vert {\phi }_{1,2}\right\rangle$ (*n* ≠ 0) on photon B as illustrated graphically in Fig. 1a. The skyrmion states are then defined as

2 $${\left\vert {\phi }_{1}\right\rangle }_{B}={\left\vert R\right\rangle }_{B}{\left\vert {\ell }_{1}\right\rangle }_{B}+{\left\vert L\right\rangle }_{B}{\left\vert {\ell }_{2}\right\rangle }_{B}$$

3 $${\left\vert {\phi }_{2}\right\rangle }_{B}={\left\vert R\right\rangle }_{B}{\left\vert {\ell }_{2}\right\rangle }_{B}+{\left\vert L\right\rangle }_{B}{\left\vert {\ell }_{3}\right\rangle }_{B}$$

where $\left\vert \ell \right\rangle =\int_{{{\mathcal{R}}}^{2}}\left.\right\vert {\mathrm{LG}}_{\ell }({\bf{r}}){e}^{i\ell \phi }\left\vert {\bf{r}}\right\rangle {d}^{2}{\bf{r}}$, with the Laguerre-Gaussian (LG) complex field represented as LG_*ℓ*_(**r**) and *ℓ*_1_, *ℓ*_2_, *ℓ*_3_ denotes the OAM of *ℓ*_1_*ℏ*, *ℓ*_2_*ℏ*, *ℓ*_3_*ℏ* per photon respectively, with ∣*ℓ*_3_∣ > ∣*ℓ*_2_∣, ∣*ℓ*_2_∣ > ∣*ℓ*_1_∣, and *ℓ*_3_ − *ℓ*_2_ = *ℓ*_2_ − *ℓ*_1_. Here, a skyrmionic topology emerges from the non-separability between the single photon’s internal spin (polarization) and OAM degrees of freedom (DoFs). However, when measured on its own, the topology of photon B is, in fact, “unknown” as photon B is described by a mixture of skyrmion states. To uncover the topology it carries, one must make a measurement on the polarization state of photon A. The topology of the single photon can thus be treated as a non-separable property of the state, manifesting only through a non-local measurement on its entangled partner. By deliberately controlling the polarization projection on photon A, we can remotely control and switch the skyrmion topology heralded on photon B, as illustrated in Fig. 1a. With this control, photon B can be made to carry either topological number *n*_1_ or *n*_2_, each corresponding to a distinct and uniquely structured skyrmion state. Our quantum entangled state thus realizes a non-local quantum dial for topological structure.

In practice, a projection onto the basis polarization states ($\left\vert R\right\rangle ,\left\vert L\right\rangle$) for photon A heralds a particular quantum skyrmion state on photon B, either $\left\vert {\phi }_{1}\right\rangle$ or $\left\vert {\phi }_{2}\right\rangle$. These states are topologically equivalent, characterized by the same skyrmion number since *ℓ*_3_ − *ℓ*_2_ = *ℓ*_2_ − *ℓ*_1_. However, a more generalized polarization projection on photon A, $\left\vert P\right\rangle \left\langle P\right\vert$, where $\vert{P}\rangle =\,{\mathrm{cos}}\,(\theta /2)\vert{R}\rangle +\,{\mathrm{sin}}\,(\theta /2){e}^{i\alpha }\vert{L}\rangle$ and *θ* ∈ [0, *π*], *α* ∈ [0, 2*π*], results in the new state

4 $${\left\vert \Psi \right\rangle }_{B| A}=\,{\mathrm{cos}}\,(\theta /2){\left\vert {\phi }_{1}\right\rangle }_{B}+\,{\mathrm{sin}}\,(\theta /2){e}^{-i\alpha}{\left\vert {\phi }_{2}\right\rangle }_{B}$$

By adjusting the relative amplitude (via *θ*) and phase (*α*) tuning angles, the polarization projection on photon A is varied, collapsing the state of photon B into either a single skyrmion state or superpositions thereof. As depicted in Fig. 1b, the superposition states define a unique topology, revealing a particular topological number at the equator of the sphere, while the single skyrmion states at the poles contribute an entirely different number, transforming the sphere into a map of topology—a topological Bloch sphere—where the basis vectors are themselves topological states. The topological Bloch sphere hence represents the state space of the skyrmion-photon, parametrized by measurements on the spin-photon, with each point on the sphere representing different Bell states of the basis skyrmion states. Consequently, we can assign a skyrmion number to each point on the sphere, providing an additional topological layer to the traditional Bloch sphere.

Traversing this topological landscape and visualizing the polarization fields at different points on the sphere reveals the underlying structures that give rise to the topological transition of photon B. For an example state with *ℓ*_1,2,3_ = 0, −2, −4, these structures are visualized in Fig. 1b. At the north pole of the sphere, the polarization texture represents a higher-order skyrmionic structure, yielding a skyrmion number of *n*_1_ = −2. However, for the superposition states off the poles, a multiskyrmion structure emerges, revealing quasiparticle-like distributions, where the quasiparticles possess individual skyrmion numbers, as shown in Fig. 1b. The total skyrmion number is found by a sum of the individual localized numbers *m*_*j*_, as *n* = ∑_*j*_*m*_*j*_, giving a wrapping number of *n*_2_ = −4 for the superposition states in our example. Importantly, the intermediate superposition states on the sphere are also described by the same skyrmion number as the equator states, i.e., *n*_2_. The skyrmion number for photon B is thus parametrized by the non-local measurement angles (*θ*, *α*) of photon A,

5 $$n(\theta ,\alpha )=\frac{1}{4\pi }\int_{{{\mathcal{R}}}^{2}}{\epsilon }_{ijk}{S}_{i}(\theta ,\alpha )\frac{\partial {S}_{j}(\theta ,\alpha )}{\partial x}\frac{\partial {S}_{k}(\theta ,\alpha )}{\partial y}dxdy$$

where *ϵ*_*ijk*_ is the Levi-Civita symbol and *S*_*i*,*j*,*k*_ = *S*_1,2,3_ are the local normalized spatially varying Stokes parameters where $\mathop{\sum }\nolimits_{i = 1}^{3}{S}_{i}^{2}=1$, ensuring a mapping to the unit sphere.

**Fig. 2:**
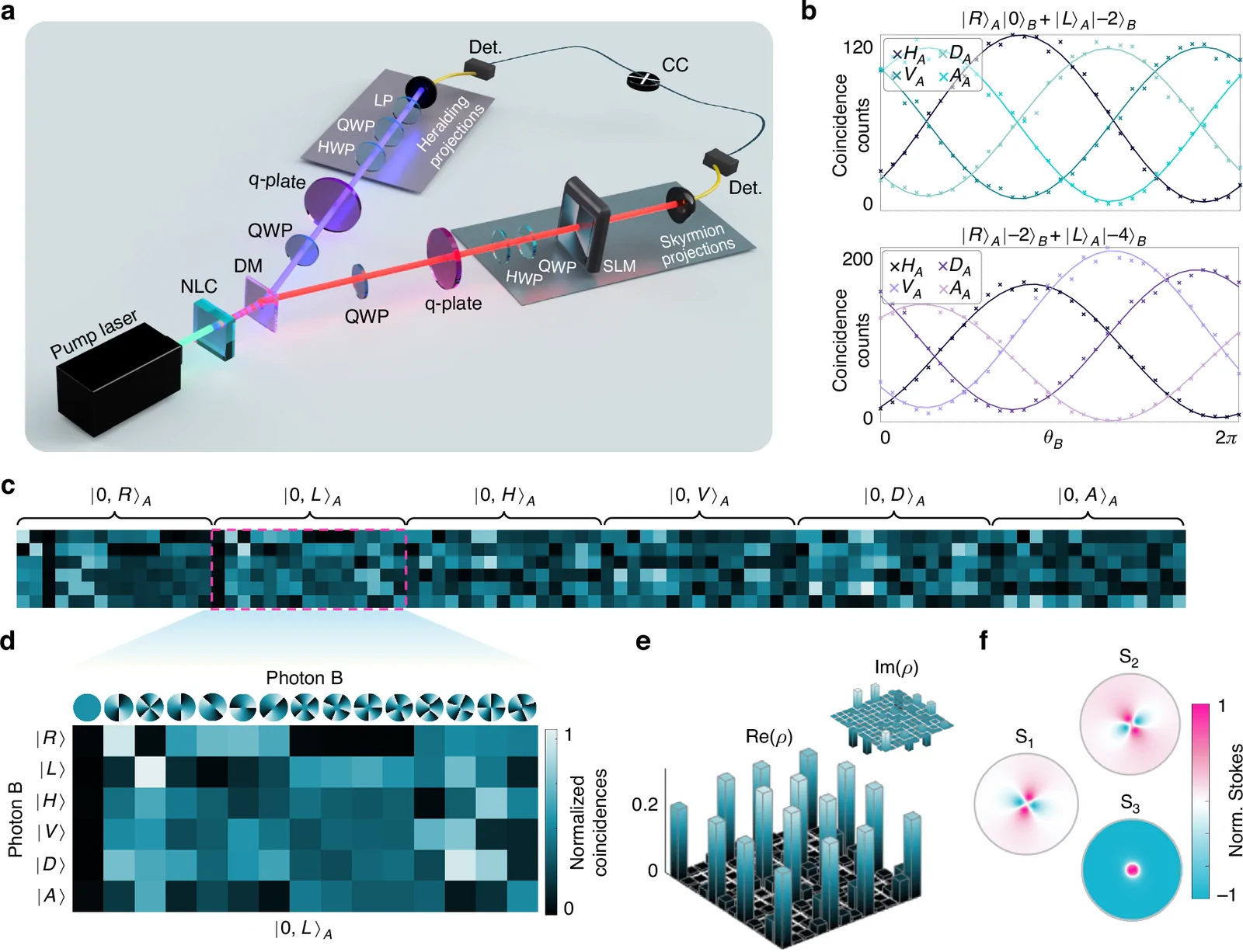
Experimental generation of spin-skyrmion entangled states. **a** Schematic diagram of the non-degenerate quantum experiment used to generate and detect spin-skyrmion entangled states. A non-linear crystal (NLC) is used to produce dual-wavelength SPDC at *λ*_*A*_ = 1550 nm and *λ*_*B*_ = 810 nm. A dichroic mirror (DM) separates the wavelengths into two arms, with a q-plate and polarization optics (quarter- and half-wave plates (QWP and HWP)) in each path. The skyrmion path is directed to an SLM for spatial and linear polarization projections, while the heralding photon is sent to a linear polarizer (LP). Both photon paths are thereafter coupled into SMFs connected to single-photon detectors. Coincidences are recorded by a coincidence counter (CC) within a 0.5 ns window. A linear polarizer is shown here for simplicity, but an SLM was used on both photons for the polarization projections. To confirm the non-local nature of the states we generate, we demonstrate a **b**, violation of the Bell inequality for an example state where *ℓ*_1_ = 0, *ℓ*_2_ = −2 and *ℓ*_3_ = −4, performing polarization projections on photon A and OAM projections on photon B. **c** The measurement matrix for the QST performed on the example state, constructed by six polarization measurements on photon A, and 15 spatial measurements coupled with six polarization measurements on photon B, shown in the zoomed-in insets in (**d**). **e** Corresponding experimentally reconstructed density matrix extracted from the full QST with real and imaginary components. **f** Stokes measurements retrieved directly from the tomography data

To verify the non-local topological control, we use the experimental setup depicted in Fig. 2a. Dual-wavelength photon pairs entangled in the spatial (OAM) degree of freedom are generated in a Type 0, non-linear crystal of length *l* = 5 mm, via spontaneous parametric downconversion (SPDC). The photon pairs are separated using a dichroic mirror, reflecting photons of wavelength *λ*_*A*_ = 1550 nm and transmitting photons of wavelength *λ*_*B*_ = 810 nm. In each arm, the crystal plane was imaged to have a beam diameter of 2*w* = 0.45 mm on an electrically-tunable q-plate^36^. The q-plates have azimuthally-varying optical axes in the transverse plane with a topological charge *q* which imparts −2*qℏ* (2*qℏ*) OAM per photon for right (left) circularly polarized light at full conversion. With half-tuning, the transformation ${\hat{U}}_{QP}\left\vert R,\ell \right\rangle \to \frac{1}{\sqrt{2}}(\left\vert R,\ell \right\rangle +\left\vert L,\ell -2q\right\rangle )$ was performed on each photon, where ${\hat{U}}_{QP}$ represents the q-plate operator at 50% conversion efficiency. This transforms our initial OAM-entangled state to a spin-skyrmion entangled tripartite state, with details of the transformation given in SI. The accessible skyrmion sectors on the topological Bloch sphere are engineered by the q-plate transformation, with the charge tailoring the skyrmion numbers of the conditional states. The plane of the q-plate was then imaged onto a spatial light modulator (SLM) on the skyrmion-photon arm. To perform the necessary projections and state tomography, we employed waveplates and the SLM. Polarization projections were made on photon A while projecting onto the spatial mode *ℓ*_*A*_ = 0, heralding a different skyrmion state on photon B, where both polarization and OAM DoFs were measured locally. A combination of a quarter-wave plate, half-wave plate, and linear polarizer was used to project onto various polarization states. Due to the polarization sensitivity of SLMs, the light emerging from the SLM was horizontally polarized, allowing the SLM to serve as a filter for the polarization projections. The half-wave plate was rotated to access linear polarization states, and the quarter-wave plate enabled projection onto circular polarization states. After the SLM and polariser, the photons were coupled to single-mode optical fibers (SMFs) connected to avalanche photo-diodes (APDs) for photon detection. The photon counting device (CC) measured the coincidences within a 0.5 ns detection window.

To confirm the non-local nature of the state with *ℓ*_1,2,3_ = 0, −2, −4, in this experiment (created using q-plates with *q* = 1), we performed measurements to demonstrate a violation of the Clauser-Horne-Shimony-Holt (CHSH) Bell inequality. Joint projective measurements were carried out between photons A and B of our quantum state, by performing polarization measurements on photon A and OAM projections on photon B for the basis skyrmion states. The measurements were carried out by selecting the polarization settings {*H*, *V*, *D*, *A*} on photon A and rotating the angle (*θ*_*B*_) of the spatial analyzer $\left\vert {\theta }_{B}\right\rangle =\left\vert 0\right\rangle +{e}^{-i{\theta }_{B}}\left\vert -2\right\rangle$ for each setting in the $\left\vert R\right\rangle$ subspace of photon B as well as $\left\vert {\theta }_{B}\right\rangle =\left\vert -2\right\rangle +{e}^{-i{\theta }_{B}}\left\vert -4\right\rangle$ in the $\left\vert L\right\rangle$ subspace. Here, H, V, D and A, respectively mean horizontal, vertical, diagonal and antidiagonal polarization states. Our results demonstrate a violation of the Bell inequality with a Bell parameter of *S* = 2.53 ± 0.005 for the heralded state ${\left\vert R\right\rangle }_{A}{\left\vert 0\right\rangle }_{B}+{\left\vert L\right\rangle }_{A}{\left\vert -2\right\rangle }_{B}$ and *S* = 2.47 ± 0.004 for state ${\left\vert R\right\rangle }_{A}{\left\vert -2\right\rangle }_{B}+{\left\vert L\right\rangle }_{A}{\left\vert -4\right\rangle }_{B}$, with Bell curves shown in Fig. 2b.

We demonstrate non-local switching of the generated local topology by performing a full quantum state tomography (QST) on the entangled photon state, following a similar procedure to ref. ^37^. For our state, this corresponds to six polarization projections on the heralding photon, and 15 spatial projections coupled with 6 polarization projections on photon B, resulting in an overcomplete QST with 540 entries shown in Fig. 2c. The density matrix (Fig. 2e) is then reconstructed with a fidelity *F* = 0.93 and purity *γ* = 0.96. Details of the density matrix construction from these measurements and of computing the corresponding entanglement witnesses is outlined in SI. To analyze the skyrmionic structure of our state, the Stokes parameters (Fig. 2f) are extracted from the density matrix and the skyrmion number is computed using Eq. (5), details of which are provided in SI.

**Fig. 3:**
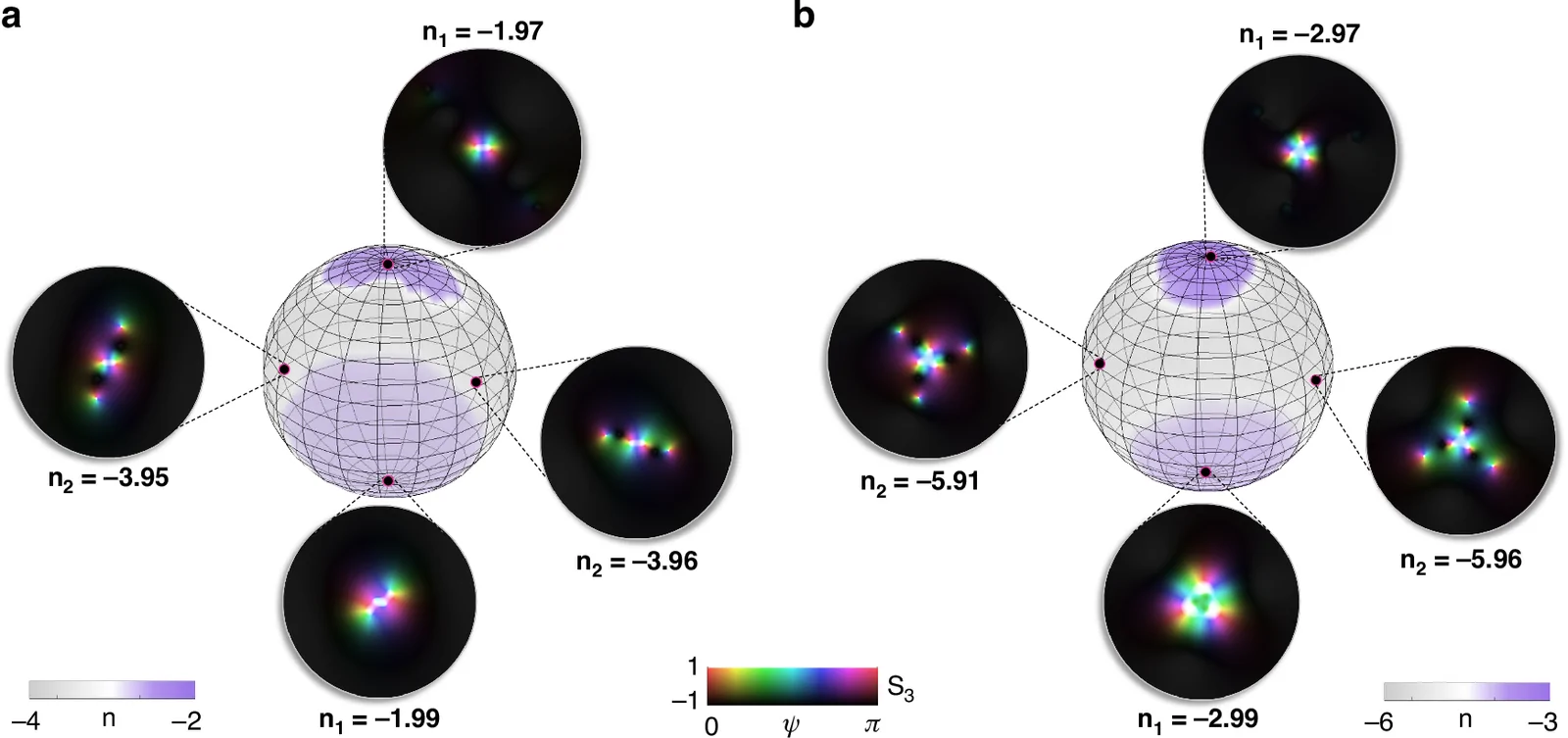
Experimental verification of non-locally controlled topology. A switch in the topological skyrmion number of photon B is realized through non-local polarization measurements on photon A. The spin textures are illustrated at different points on the sphere for a state with **a**, *ℓ*_1_ = 0, *ℓ*_2_ = −2 and *ℓ*_3_ = −4, yielding a switching of the skyrmion number from *n*_1_ = −2 at the poles to *n*_2_ = −4 at the equator and **b**, *ℓ*_1_ = 0, *ℓ*_2_ = −3 and *ℓ*_3_ = −6, switching between the two distinct skyrmion numbers, *n*_1_ = −3 and *n*_2_ = −6

Figure 3 represents the experimental verification of the non-local topological switch for two example states, where *ℓ*_1,2,3_ = 0, −2, −4 (Fig. 3a) and *ℓ*_1,2,3_ = 0, −3, −6 (Fig. 3b). Here, the latter state was generated using q-plates with *q* = 1.5. The spin textures are visualized at different points on the topological sphere in Fig. 3, representing the skyrmionic structure of our state. In Fig. 3a, a skyrmion number of *n*_1_ ≈ −2 is observed at the poles of the sphere, whereas a multiskrymion structure is realized at the equator, yielding *n*_2_ ≈ −4. In particular, we obtain a skyrmion number of *n*_1_ = −1.97 ± 0.02 and *n*_1_ = −1.99 ± 0.002 at the poles of the sphere, as well as *n*_2_ = −3.95 ± 0.004 and *n*_2_ = −3.96 ± 0.002 for the example states shown. Similar behavior is observed for the state in Fig. 3b, where the number of quasiparticles in the multiskyrmion structure now increases to three, producing a total skyrmion number of *n*_2_ ≈ −6 at the equator, whereas the higher-order skyrmion at the poles has a wrapping number of *n*_1_ ≈ −3. For the examples shown in Fig. 3b, we obtain a skyrmion number of *n*_1_ = −2.97 ± 0.011 and *n*_1_ = −2.99 ± 0.002 at the poles of the sphere, as well as *n*_2_ = −5.91 ± 0.007 and *n*_2_ = −5.96 ± 0.011 for the example states shown. Notice that the portion of the spheres corresponding to the basis skyrmion states extends beyond just the polar regions, due to experimental crosstalk in the OAM subspaces measured. This spread is more confined for the *ℓ*_1,2,3_ = 0, −3, −6 subspace where the wider separation in OAM values minimizes crosstalk effects.

The retrieved skyrmion numbers are consequently influenced by the purity of the reconstructed state, which can be degraded by experimental imperfections such as the aforementioned OAM mode crosstalk, or even imperfect OAM and polarization projections. For the OAM projections, the coupling efficiencies of different modes into the single-mode fibers can result in uneven and inaccurate modal contributions. This was mitigated by a careful choice of the beam size on the encoded projection holograms, together with precise adjustment of the physical beam size coupled into the SMF. For the polarization projections, potential sources of errors could be imperfections in the alignment (imprecise waveplate angles) and performances of the polarization optics such as waveplates and polarizers, which can be mitigated by repeating the experiment several times. Additionally, we see a small deviation in the experimentally computed skyrmion numbers and the theoretically expected values. This is caused by a common source of error in most systems, where the limited region of interest (ROI) in the detection space, and the finite resolution describing the field (discussed in detail in ref.^38^), results in small deviations from theory. In our particular case of the multiskyrmions, an additional source of error arises from the ability to resolve and detect the localized quasiparticles as they are moving in and out of the detection window. To mitigate this error, the beam sizes of the extracted Stokes parameters were chosen judiciously to ensure that for a small relative amplitude (*θ*) value, the quasiparticles do not completely fly away to infinity, but are still large enough to be resolved for large *θ*.

These results experimentally confirm the non-local influence of photon A on the local topological structure of photon B, realizing a non-local skyrmion number switch. Our experimental results also confirm the expected multiskyrmion structure at the equator for both cases. While these results demonstrate a binary switch (between two different skyrmion numbers), we can extend this further by varying the type of heralded projection made on photon A, producing a ternary switch (between three distinct topological numbers), and also easily extending our skyrmion structures to higher-order skyrmions and multiskyrmions with more quasiparticles. This extended case is described in detail in the SI, accompanied by numerical simulations that confirm a ternary switch.

### Quantum multiskyrmion

**Fig. 4:**
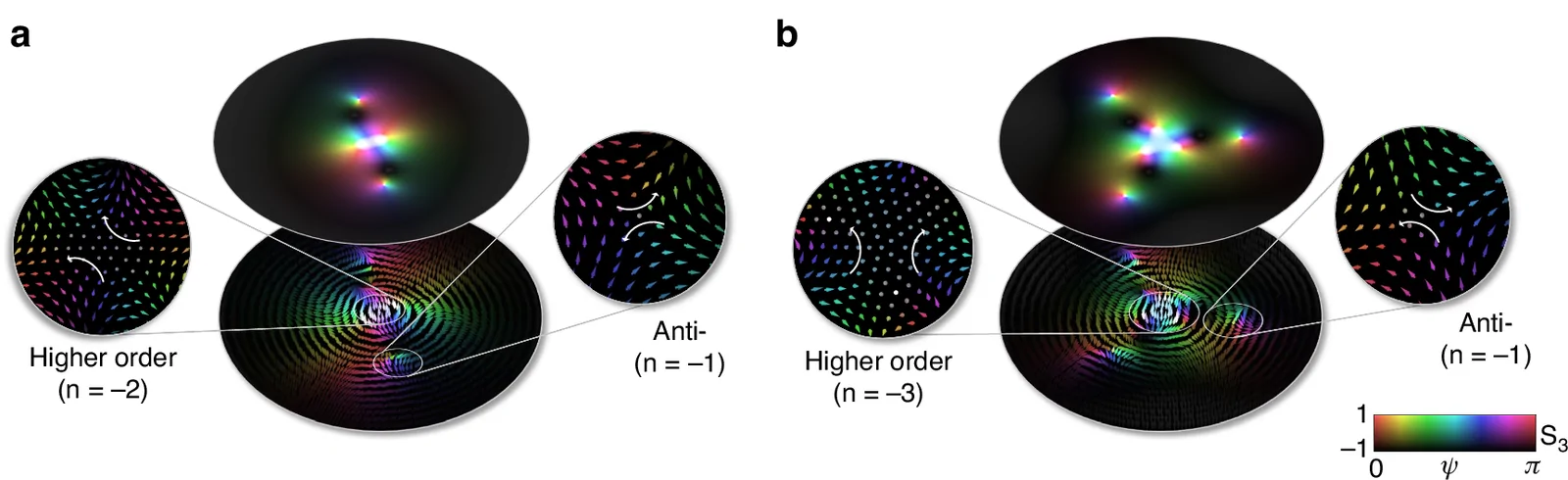
Experimental quantum multiskyrmions. Spin textures of the different realized quantum multiskyrmion flavours, with zoom-in insets revealing the local quasiparticle polarization textures embedded in the structure for a state with **a,** *ℓ*_1_ = 0, *ℓ*_2_ = −2 and *ℓ*_3_ = −4, and **b,** *ℓ*_1_ = 0, *ℓ*_2_ = −3 and *ℓ*_3_ = −6

Thus far, we have studied how the topology of photon B is affected by a measurement on photon A, whilst remaining agnostic to photon B’s “local" polarization texture. We now instead look directly at the quantum multiskyrmion structure revealed via non-local control. Figure 4 shows a visualization of the spin texture of the two flavors of quantum multiskyrmions created in our experiment. The polarization texture of the multiskyrmion reveals distinct local textures embedded within the broader field. Zooming into the individual skyrmions shows the hyperbolic polarization texture characteristic of the anti-skyrmion for the quasiparticle in each case, and the expected signature with increased vorticity is seen for the central higher-order skyrmion^11^. Note that our scheme is not restricted to the aforementioned skyrmion and multiskyrmion textures, but can be readily extended to a broader class of textures even within the multiskyrmion structure. This is achieved by simply modifying the input polarization incident on the q-plates, which enables tuning from negative to positive skyrmion numbers and generates distinct localized skyrmion textures, such as Néel, Bloch, and intermediate-type, within a single multiskyrmion structure. These variations are illustrated in the simulations presented in the SI for the ternary switch case.

### Non-locally derived quasiparticle dynamics

**Fig. 5:**
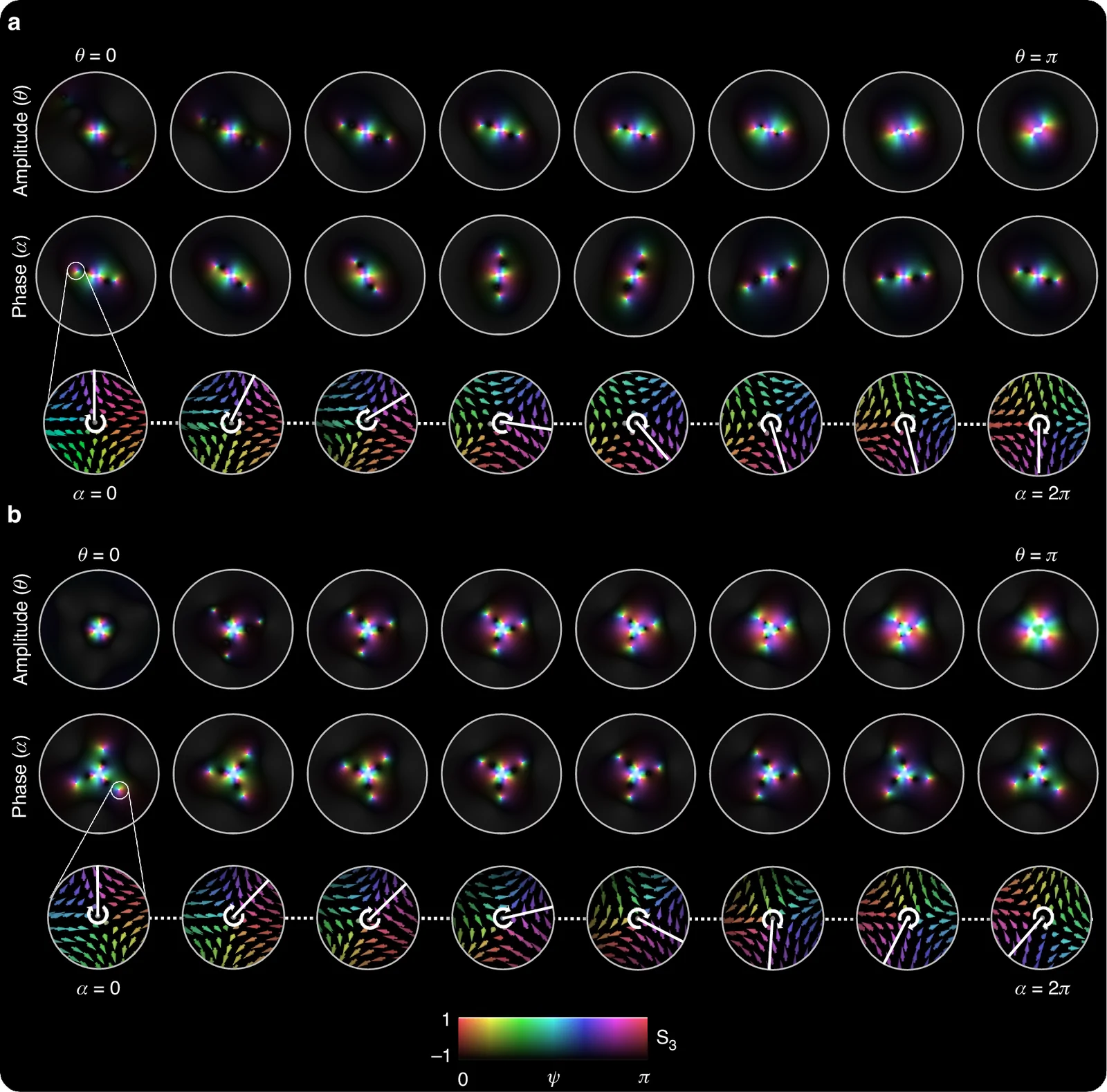
Quasiparticle dynamics. Non-locally derived polarization textures where the localized quasiparticle skyrmion vector distributions shift in and out radially when varying *θ* for *α* fixed at 3.77 rad from left to right, or exhibit an orbital and spin motion when varying *α* for *θ* fixed at 1.26 rad, for the state with **a,** *ℓ*_1_ = 0, *ℓ*_2_ = −2 and *ℓ*_3_ = −4, and for the state with **b**, *ℓ*_1_ = 0, *ℓ*_2_ = −3 and *ℓ*_3_ = −6. In **a**, the amplitude (*θ*) varies from left to right as *θ* = [0, 0.35, 0.69, 1.05, 1.75, 2.09, 2.44, 3.14] rad and the phase (*α*) as *α* = [0, 0.69, 1.40, 2.79, 4.19, 4.89, 5.59, 6.28] rad and in **b**, *θ* = [0, 0.69, 1.05, 1.40, 1.75, 2.09, 2.79, 3.14] rad and *α* = [0, 1.40, 2.09, 2.79, 3.49, 4.19, 4.89, 6.28] rad

Having verified the existence of a multiskyrmion structure with localized quasiparticles, we are now interested in studying the dynamics of the polarization texture of photon B as we incrementally change the projection angles on photon A. In Fig. 5, the quasiparticle distributions of photon B’s skyrmion state are shown as the amplitude, *θ*, and phase, *α*, are varied continuously. Results for the state with OAM values *ℓ*_1,2,3_ = 0, −2, −4 and *ℓ*_1,2,3_ = 0, −3, −6 are shown in Figs. 5a and b, respectively. A radial motion of the quasiparticles is observed when varying the amplitude, with the quasiparticles approaching the central distribution from infinity as *θ* increases from 0 to *π*. This behavior is mimicked in both sets of results, with the only difference being in the number of quasiparticles experiencing these dynamics: 2 and 3 quasiparticles for Fig. 5a and Fig. 5b, respectively. For *θ* angles close to *π*, the quasiparticles start to merge with the central distribution, inhibiting the ability to detect them as separate localized distributions. The example spin textures illustrated for varying *θ* correspond to states located at the poles of the Bloch sphere, as well as intermediate superposition states. Varying the phase (*α*) results in the orbit of the quasiparticles about the central distribution with increasing *α* from 0 to 2*π*. In addition to the orbiting behavior, the quasiparticles also exhibit a local spin, which is represented in the insets showing the evolution of the selected quasiparticle as the rotation of the phase profile *ψ*. This is induced by a local precession of each Stokes vector about the *S*_3_ axis. Whilst this behavior is also mimicked in both sets of results, the symmetry of the quasiparticle distribution plays a role in the total spin and orbit angles performed by each quasiparticle within the distribution. Under a full scan of phase projection angles *α*, the quasiparticles in Fig. 5a perform an orbit and spin of *π* and in Fig. 5b exhibit an orbit of $\frac{2\pi }{3}$ and spin of $\frac{4\pi }{3}$, which is consistent with the 2- and 3-fold symmetries of their respective distributions.

### Embedded GHZ state and topological structure

**Fig. 6:**
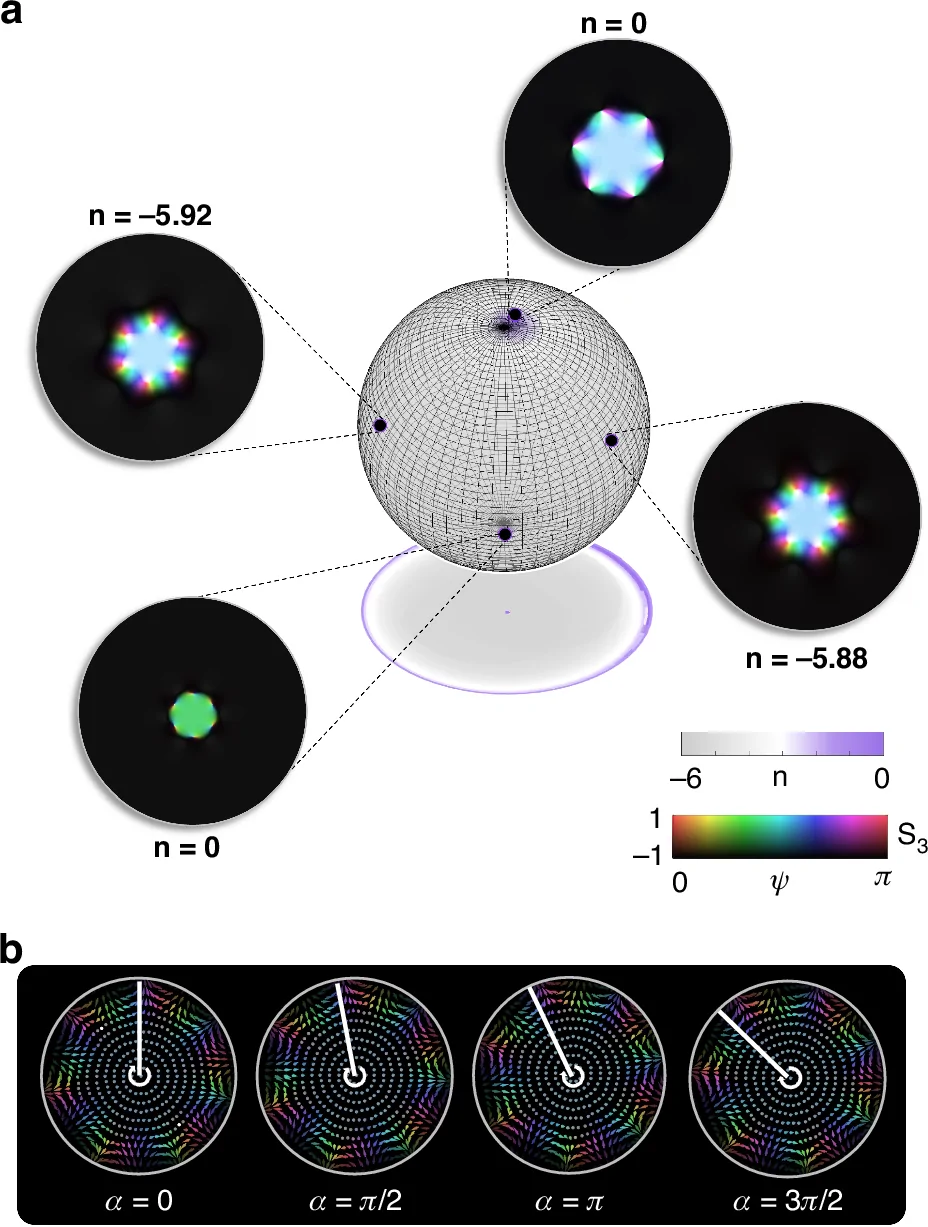
Topology of the embedded GHZ-like states. **a** Topological landscape encoded across photon A’s polarization as well as photon B’s OAM and polarization degrees of freedom. Basis projections on photon A select separable spin–OAM states, giving trivial topology (*n* = 0) at the poles, while superposition projections reveal the GHZ-to-Bell signature as skyrmionic textures with *n* = −6 at the equator. **b** Spin textures of the four equatorial Bell-like states. Each carries the same skyrmion number, but has a distinct texture, making the Bell-state structure visible through the topology of photon B

Next, we show that a GHZ-like component can be isolated from our tripartite state, revealing how a characteristic signature of multipartite entanglement can be visualized through topology. Here, a superposition projection on one subsystem of a GHZ state projects the remaining two into a Bell state. In the present realization, this Bell state is encoded in the spin-OAM degrees of freedom of photon B, providing a direct topological visualization of this effect; the Bell states are thus realized as skyrmions with transforming spin textures as illustrated in Fig. 6a, which can be smoothly deformed into quasiparticle systems with more complex dynamics.

To see this, we remind the reader of the previous section, where a polarization measurement on photon A collapsed the state of photon B into a non-separable state exhibiting correlations between its internal spatial and polarization DoFs, producing complex orbital and spin dynamics. This is indeed reminiscent of the aforementioned tripartite GHZ state, which collapses to an entangled Bell state upon a similar projective measurement. Specifically, by constraining photon B to the spatial Hilbert space spanned by the OAM states $\left\vert {\ell }_{1}\right\rangle$ and $\left\vert {\ell }_{3}\right\rangle$, we can extract the following GHZ-like state

6 $${\left\vert {\Psi }_{{\rm{GHZ}}}\right\rangle }_{AB}=\frac{1}{\sqrt{2}}\left({\left\vert R\right\rangle }_{A}{\left\vert R\right\rangle }_{B}{\left\vert {\ell }_{1}\right\rangle }_{B}+{\left\vert L\right\rangle }_{A}{\left\vert L\right\rangle }_{B}{\left\vert {\ell }_{3}\right\rangle }_{B}\right)$$

where, by identifying the logical qubit basis $({\left\vert R\right\rangle }_{A},{\left\vert L\right\rangle }_{A})\to (\left\vert 0\right\rangle ,\left\vert 1\right\rangle )$, $({\left\vert R\right\rangle }_{B},{\left\vert L\right\rangle }_{B})\to (\left\vert 0\right\rangle ,\left\vert 1\right\rangle )$, and $({\left\vert {\ell }_{1}\right\rangle }_{B},{\left\vert {\ell }_{3}\right\rangle }_{B})\to (\left\vert 0\right\rangle ,\left\vert 1\right\rangle )$, the state takes the GHZ form $(\left\vert 000\right\rangle +\left\vert 111\right\rangle )/\sqrt{2}$. As before, performing polarization measurements on photon A then reveals the topological sphere and behavior of the embedded GHZ state as shown in Fig. 6 for *ℓ*_1,2,3_ = 0, −3, −6 with Fig. 6a showing the topological space and Fig. 6b confirming the texture for the four superposition measurements (*α* = 0, *π*/2, *π*, 3*π*/2). Further details are provided in the SI. Projections onto the basis states collapse our GHZ-like state into the separable states ${\left\vert R\right\rangle }_{B}{\left\vert 0\right\rangle }_{B}$ and ${\left\vert L\right\rangle }_{B}{\left\vert -6\right\rangle }_{B}$, each carrying a trivial topology (*n* = 0) depicted at the poles of the topological sphere shown in Fig. 6a. However, projections onto the superposition states defined as ${\left\vert \alpha \right\rangle }_{A}={\left\vert R\right\rangle }_{A}+{e}^{-i\alpha }{\left\vert L\right\rangle }_{A}$ collapses our GHZ-like state into non-separable states of the form ${\left\vert R\right\rangle }_{B}\left\vert 0\right\rangle +{e}^{-i\alpha }{\left\vert L\right\rangle }_{B}\left\vert -6\right\rangle$, which is characterized by a skyrmion number *n* = −6 and is depicted at the equator of the sphere shown in Fig. 6a. The skyrmionic texture therefore provides a topological visualization of the conditional Bell-state correlations: basis projections select one correlated component, yielding trivial textures, whereas superposition projections coherently combine both components, yielding skyrmions. The four Bell states are then obtained by projecting onto states with *α* = {0, *π*, *π*/2, 3*π*/2} with their corresponding spin textures shown in Fig. 6b. Each Bell state corresponds to a different topological texture of a single central quasiparticle. Since the Bell states and the states considered in Eq. (4) (for the same polarization measurements) are characterized by identical skyrmion numbers, they can be smoothly deformed into one another. Here the smooth deformation is driven by interfering the GHZ state with a reference state ${\left\vert {\Psi }_{{\rm{ref}}}\right\rangle }_{AB}=\left({\left\vert R\right\rangle }_{A}{\left\vert L\right\rangle }_{B}+{\left\vert L\right\rangle }_{A}{\left\vert R\right\rangle }_{B}\right){\left\vert {\ell }_{2}\right\rangle }_{B}$. Therefore, the Bell state signature of the embedded GHZ state manifests physically as diverse quasiparticle dynamics derived from the localized spin-texture of photon B, given a superposition measurement on photon A. In this way, we establish an intrinsic link between the entanglement dynamics of GHZ states and the evolution of their underlying topological structure.

## Discussion

Whilst the initial driving force behind the search for skyrmionic topological features in optical systems has been spurred on by their promise as robust information carriers within magnetic-spin systems, the field has since matured into demonstrations of their resilience across various optical systems such as classical vector beams^23,39^, quantum non-local entangled states^33,39,40^ and single photon spin-orbit coupled states^35^. These studies open routes for using the topological invariants of optical systems for a robust information encoding alphabet, with a few early proof of principle demonstrations shown in optical anisotropic systems^41^ and within vector beams transmitted through random media^42^. Our work further extends this paradigm from a fundamental perspective by studying the topology embedded in more exotic quantum states of light. Quantum entangled states, in particular, present a unique set of opportunities, where the topological classification of high-dimensional and multipartite entangled states with diverse entanglement structures is almost completely unexplored.

This work extends the notion of the topological classification of quantum states to a tripartite entangled state through the introduction of a “topological Bloch sphere", highlighting the existence of multiple topological transition points, and connecting this work to recent discoveries which reveal the topological spectrum of high-dimensional entangled states^43^. Our tripartite state, in particular, allows up to three topological symbols without scaling the dimensionality of the state space beyond two dimensions, suggesting a route towards higher capacity information encoding by exploiting state complexity within multipartite states.

By engineering exotic quantum states, our work reveals an intrinsic link between the entanglement dynamics of tripartite states and skyrmion topologies. We thus extend the notion of visualizing entanglement^44,45^ by leveraging topological quasiparticles and thereby pave the way for the physical observation of multipartite state evolution. In this case, rather than remotely controlling the quasiparticle dynamics, one can use the evolving skyrmionic structure of one photon as a probe to infer the effect of a perturbation on its entangled partner. Specifically, by keeping the measurement on the spin photon fixed, any observed changes in the system’s dynamics can then be attributed purely to external perturbations. The effect of these perturbations may manifest either as transitions between topological sectors on the topological Bloch sphere or as more finer effects on the localized quasiparticle structures. Our tripartite states, therefore, manifest distinct topological and quasiparticle features which may be used to uniquely sense complex channel information, opening exciting possibilities for quantum topological sensing.

Furthermore, while previous studies have considered topology only as a predefined property of the state, we engineered quantum states in which topology emerges as a non-separable feature of the system, enabling remotely engineered skyrmionic topologies and building upon recent work on remotely controlled topological systems^46^. This paves the way towards the transmission of this robust resource within a quantum network, granting one party access to topological structures that would otherwise be inaccessible due to limited experimental capabilities. By simply sharing one photon with a resource-scarce partner, complex skyrmions with rich internal structure and dynamics, exceeding even dual topological switches (see SI), can be remotely transmitted for use as non-classical resources in quantum applications, where robustness to noise and high-dimensional structures are advantageous. We therefore envision our work driving new research avenues focused on identifying and exploiting the complex topological structures of high-dimensional and multipartite states.

In conclusion, we have presented a unique realization of quantum topology in the form of spin-skyrmion entangled states, realizing a remotely controlled topological switch between distinct topological states. Through this experimental verification, we emulate structures seen in magnetic systems, realizing the first quantum multiskyrmions reminiscent of magnetic biskyrmions^47^, with our non-local control enabling us to replicate the spin and orbital dynamics of the quasiparticles, advancing beyond static optical skyrmions to non-locally tunable quantum multiskyrmions with quasiparticle dynamics. The topological states we create contain embedded GHZ-like states with tripartite entanglement dynamics manifesting physically as evolving topological quasiparticle structures. While our approach is compatible with degenerate quantum-entangled systems, our experimental realization in a non-degenerate entanglement setup extends the versatility of the approach to dual-wavelength systems, allowing topological control and detection to be implemented across configurations benefiting from interactions at different wavelengths. For instance, non-linear quantum transport^48^ of the topological states, remote controlled transfer of the skyrmionic topology to matter^27^, or topological interaction at advantageous wavelengths, while controlled with technologically accessible ones^49–51^. We expect these findings to open new avenues for harnessing and dynamically manipulating quantum skyrmions and multiskyrmions for multi-level quantum information encoding protocols and quantum topological sensing of evolving systems.

## Materials and methods

### Density matrix reconstruction

To reconstruct the density matrix describing the quantum state of the bipartite system, we perform a quantum state tomography (QST) in the composite Hilbert space ${\mathcal{H}}={{\mathcal{H}}}_{{\rm{pol,A}}}\otimes {{\mathcal{H}}}_{{\rm{pol,B}}}\otimes {{\mathcal{H}}}_{{\rm{sp}},{\rm{B}}}$. Here, photon A is characterized solely by its polarization, while photon B is described by both polarization and spatial degrees of freedom. The density matrix *ρ* can be expanded in a complete operator basis

7 $$\rho =\frac{1}{d}\sum _{i,j,k}{c}_{ijk}({\sigma }_{i}\otimes {\sigma }_{j}\otimes {\lambda }_{k})$$

where *d* = *d*_pol,A_ × *d*_pol,B_ × *d*_sp,B_ is the total Hilbert space dimension, {*σ*_*i*_} and {*σ*_*j*_} respectively denote the Pauli operator basis for the polarization degrees of freedom of photons A and B, {*λ*_*k*_} are the generalized Gell-Mann operators spanning the spatial subspace of photon B, and *c*_*ijk*_ are the state coefficients. We construct an informationally complete set of measurements from tensor products of the projective measurements on each subsystem. The polarization measurements for both photons use eigenprojectors of the Pauli operators, whereas the spatial measurements on photon B use eigenprojectors of the generalized Gell-Mann operators. Each measurement setting is represented by a projector of the form ${M}_{k}={P}_{a}^{(A,{\rm{pol}})}\otimes {P}_{b}^{(B,{\rm{pol}})}\otimes {P}_{s}^{(B,{\rm{sp}})}$ and the corresponding detection probability is given by the Born rule, ${p}_{k}={\rm{Tr}}(\rho {M}_{k})$. The reconstruction proceeds as a linear inversion problem. After vectorizing both the density matrix and the measurement operators, the forward model takes the form **p** = *T* vec(*ρ*), where *T* is the measurement matrix assembled from all projectors. A physically valid estimate of *ρ* is obtained by minimizing the least-squared norm of the residual

8 $${\left\vert {\bf{p}}-T{\rm{vec}}(\rho )\right\vert }_{2}^{2}$$

between the experimentally measured probabilities and the Born-rule predictions. We enforce Hermiticity, positivity, and unit trace by parametrizing the density matrix in the form $\rho =\frac{L{L}^{\dagger }}{{\rm{Tr}}(L{L}^{\dagger })}$ where *L* is a complex lower-triangular matrix with real exponential diagonal entries. This parametrization guarantees positive semi-definiteness throughout the optimization.

### Purity and fidelity

The purity, *γ*, of states provides an estimate of the total signal-to-noise ratio (SNR) within a given experiment^52^. Furthermore, the purity quantifies the degree of mixture of a state given by

9 $$\gamma =\,\mathrm{Tr}\,\left({\rho }^{2}\right)\ge \frac{1}{d}$$

where *γ* = 1 indicates a pure state and $\gamma =\frac{1}{d}$ indicates a maximally mixed state. For the states considered in this work, *γ* scales with the dimension of the spatial qudit such that $\gamma =\frac{1}{4{d}_{sp,B}}$.

The fidelity also served as an entanglement witness for our states, used to analytically compare our measured state, *ρ* against the closest, pure maximally entangled state ${\rho }_{T}=\left\vert \Psi \right\rangle \left\langle \Psi \right\vert$,

10 $$F={\left(\mathrm{Tr}\left(\sqrt{\sqrt{{\rho }_{T}}\rho \sqrt{{\rho }_{T}}}\right)\right)}^{2}$$

The fidelity is 0 if the states are orthogonal or 1 when they are identical up to a global phase.

## Supplementary information

## Data Availability

Data supporting this study are available from the authors upon reasonable request.
