# Supplementary material for: Remote engineering of particle-like topologies to visualise entanglement dynamics
Source: Light Sci Appl. 2026 Sep 2;15:368. doi: 10.1038/s41377-026-02443-x (PMC13538466; doi:10.1038/s41377-026-02443-x)
Supplement: Supplementary file 1 [file 41377_2026_2443_MOESM1_ESM.pdf]

Supplementary information for: Remote engineering of particle-like topologies to visualise entanglement dynamics

Fazilah Nothlawala,[1] Bereneice Sephton,[2,*] Pedro Ornelas,[1] Mwezi Koni,[1] Bruno Piccirillo,[2] Liang Feng,[3] Isaac Nape,[1] Vincenzo D'Ambrosio,[2] and Andrew Forbes[1]

[1]*School of Physics, University of the Witwatersrand, Private Bag 3, Wits 2050, South Africa*
[2]*Dipartimento di Fisica, Università di Napoli Federico II, Complesso Universitario di Monte S. Angelo, Via Cintia, 80126 Napoli, Italy*
[3]*Department of Electrical and Systems Engineering, University of Pennsylvania, Philadelphia, PA 19104, USA*

**Corresponding author: bereneice.sephton@unina.it*

NON-LOCAL TOPOLOGICAL CONTROL WITH ARBITRARY ℓ_A PROJECTIONS ON PHOTON A

Here we consider the OAM-entangled SPDC state following the insertion of identical half-tuned q-plates, with charge q in both arms, and examine the effect of arbitrary OAM projections on the heralding photon. We begin with a general OAM entangled state (assuming R polarization)

$$|\Psi\rangle = \sum_{\ell\in\mathbb{Z}} c_{\ell,-\ell} \, |\ell\rangle_A \, |R\rangle_A |{-\ell}\rangle_B \, |\mathrm{R}\rangle_B \,, \tag{S1}$$

where $c_{\ell,-\ell}$ is the the two-photon probability amplitude. The half-tuned q-plate transformation, $\hat{U}_{QP}\,|R,\ell\rangle \rightarrow \frac{1}{\sqrt{2}}(|R,\ell\rangle + |L,\ell-2q\rangle)$ with $\hat{U}_{QP}$ representing the q-plate operator at 50% conversion efficiency, is applied on both photons producing

$$|\Psi\rangle = \frac{1}{2}\sum_{\ell\in\mathbb{Z}} c_{\ell,-\ell}\,\{|\ell\rangle_A\,|R\rangle_A\,(|R\rangle_B\,|{-\ell}\rangle_B + |L\rangle_B\,|{-\ell}-2q\rangle_B) + |\ell-2q\rangle_A\,|L\rangle_A\,(|R\rangle_B\,|{-\ell}\rangle_B + |L\rangle_B\,|{-\ell}-2q\rangle_B)\}\,, \tag{S2}$$

For a projection of photon A onto the fundamental Gaussian mode, $\ell_A = 0$, we create the spin-skyrmion entangled states presented in the main text with simulated results for the cases with $\ell_{1,2,3} = 0, -2, -4$ and $\ell_{1,2,3} = 0, -3, -6$ represented in Fig. S1, showing excellent agreement with the binary topological switch seen in the experimental results. The polarization distribution plots shown here correspond to different regions of the sphere for the superposition states, while still yielding a total skyrmion number of $n \approx -4$ and $n \approx -6$ for these states. Notably, the distribution on the sphere corresponding to $n = -2$ and $n = -3$ is more confined to the polar regions compared to the experimental results which are affected by crosstalk. Moreover, a finer sampling of the relative amplitude (θ) and phase (ϕ) angles would also produce a topological sphere with more localized polar regions.

In the previous simulations and the experimental realization of this work, we considered the case where photon A was projected onto the mode $\ell_A = 0$. This projection, however, need not be confined to the fundamental mode in order to achieve a state in the form of Eq. 1 (main text), but can in principle be extended to any arbitrary value. Given a projection of photon A onto any arbitrary OAM mode, such as ℓ'_A, the quantum state becomes

$$\begin{aligned} |\Psi'\rangle &= \langle\ell'_A|_A\,|\Psi\rangle \\ &\propto \sum_{\ell\in\mathbb{Z}} \{\langle\ell'_A|\ell|\ell'_A|\ell\rangle_A\,|R\rangle_A\,(|R\rangle_B\,|{-\ell}\rangle_B + |L\rangle_B\,|{-\ell}-2q\rangle_B) + \langle\ell'_A|\ell-2q|\ell'_A|\ell-2q\rangle_A\,|L\rangle_A\,(|R\rangle_B\,|{-\ell}\rangle_B + |L\rangle_B\,|{-\ell}-2q\rangle_B)\} \\ &\propto |R\rangle_A\,(|R\rangle_B\,|{-\ell'_A}\rangle_B + |L\rangle_B\,|{-\ell'_A}-2q\rangle_B) + |L\rangle_A\,(|R\rangle_B\,|{-\ell'_A}-2q\rangle_B + |L\rangle_B\,|{-\ell'_A}-4q\rangle_B) \end{aligned} \tag{S3}$$

with the orthogonality of OAM modes rendering terms $\langle\ell'_A|\ell|\ell'_A|\ell\rangle$ and $\langle\ell'_A|\ell-2q|\ell'_A|\ell-2q\rangle$ non-zero only when $\ell = \ell'_A$ and $\ell = \ell'_A + 2q$, respectively. It follows that photon B is conditioned onto the spin-orbit coupled states, $|\phi_1\rangle_B = |R\rangle_B\,|{-\ell'_A}\rangle_B + |L\rangle_B\,|{-\ell'_A}-2q\rangle_B$ and $|\phi_2\rangle_B = |R\rangle_B\,|{-\ell'_A}-2q\rangle_B + |L\rangle_B\,|{-\ell'_A}-4q\rangle_B$, by projection onto a single OAM mode on photon A. This is in agreement with the general form of Eqs. 1-3 (main text) where $\ell_1 = -\ell'_A$, $\ell_2 = -\ell'_A - 2q$ and $\ell_3 = -\ell'_A - 4q$ is determined by the combined choice of spatial projection on photon A as well as the charge of the chosen q-plates. The available skyrmion topologies for switching is given by $n_1 = \ell_3 - \ell_2 = \ell_2 - \ell_1 = -2q$

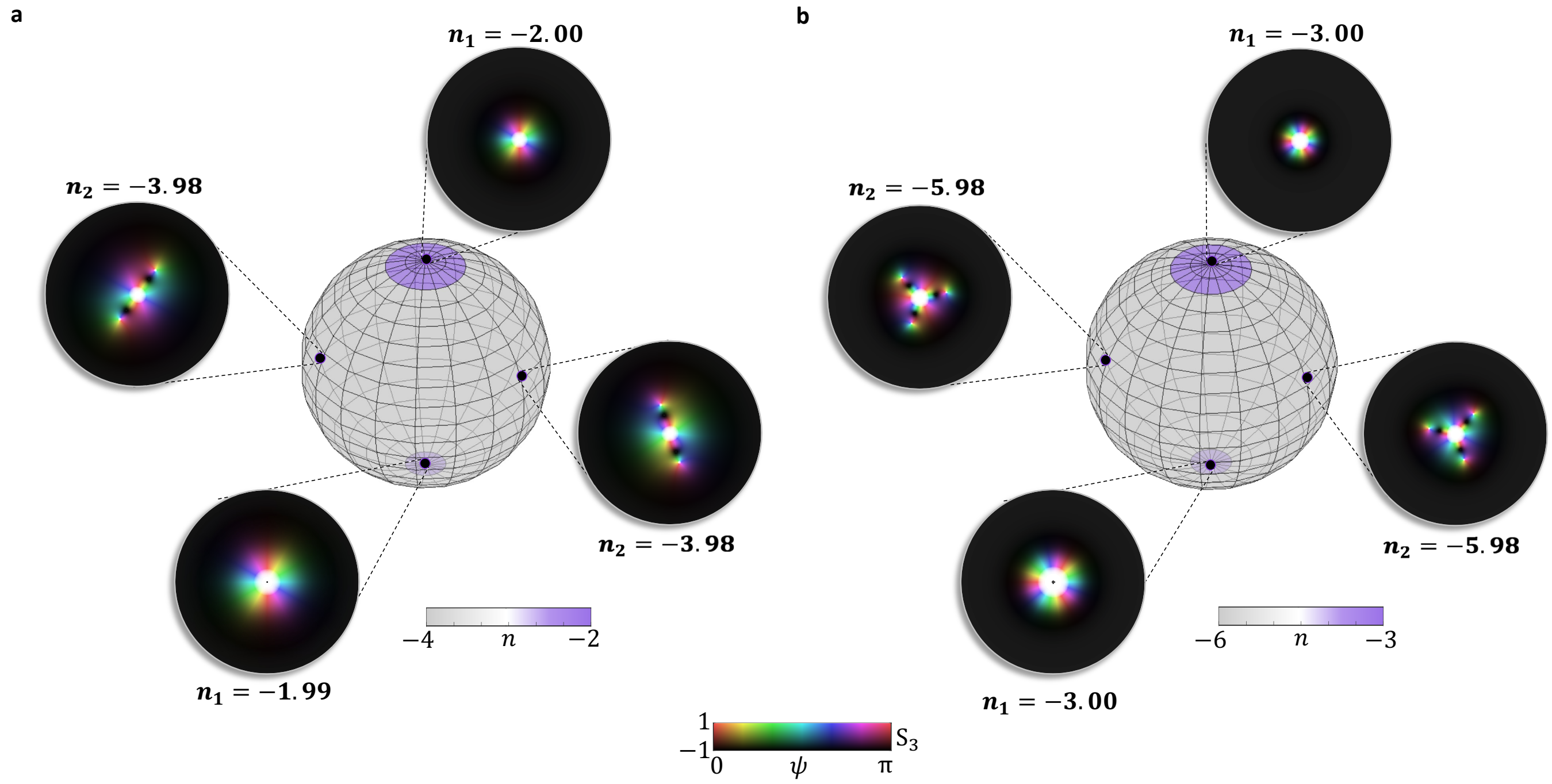

FIG. S1. **Simulated binary topology switch results.** A binary switch in the topological skyrmion number of photon B is realised, with the spin textures illustrated at different points on the sphere for a state with **a,** $\ell_{1,2,3} = 0, -2, -4$, yielding a switching of the skyrmion number from $n_1 = -2$ at the poles to $n_2 = -4$ across the remaining sphere and **b,** $\ell_{1,2,3} = 0, -3, -6$, switching between the two distinct skyrmion numbers, $n_1 = -3$ and $n_2 = -6$.

and $n_2 = \ell_3 - \ell_1 = -4q$. As a result, non-zero values of ℓ_A can be used to tailor the OAM subspaces in which the skyrmions are generated. However, it is strictly the charge of the q-plates which allows one to tailor the skyrmion numbers.

Interestingly, non-local control of extended topological transitions is possible by instead choosing to project photon A onto a superposition of OAM states. For instance, by projecting onto the two-mode superposition, $|\ell_A\rangle \rightarrow \frac{1}{\sqrt{2}}(|\ell'_{A_1}\rangle + |\ell'_{A_2}\rangle)$ where ℓ'_{A1} and ℓ'_{A2} are different OAM values, the general quantum state becomes

$$\begin{aligned} |\Psi'\rangle &= \frac{1}{\sqrt{2}}\left[\langle\ell'_{A_1}| + \langle\ell'_{A_2}|\right]|\Psi\rangle \\ &\propto |R\rangle_A\left(|R\rangle_B\left[|-\ell'_{A_1}\rangle_B + |-\ell'_{A_2}\rangle_B\right] + |L\rangle_B\left[|-\ell'_{A_1} - 2q\rangle_B + |-\ell'_{A_2} - 2q\rangle_B\right]\right) \\ &+ |L\rangle_A\left(|R\rangle_B\left[|-\ell'_{A_1} - 2q\rangle_B + |-\ell'_{A_2} - 2q\rangle_B\right] + |L\rangle_B\left[|-\ell'_{A_1} - 4q\rangle_B + |-\ell'_{A_2} - 4q\rangle_B\right]\right). \end{aligned} \tag{S4}$$

Here, each polarization term of photon B, which was previously conditioned to hold a single OAM value, is now conditioned onto a superposition of the chosen OAMs; each of which undergo the same transformation of the q-plate as before (Eq. S3). To further explore the topological transitions, we choose $\ell_{A_1} = 0$, $\ell_{A_2} = -1$ and $q = 2.5$ as an example, heralding the state

$$|\Psi'\rangle \propto |R\rangle_A\left(|R\rangle_B\left[|0\rangle_B + |1\rangle_B\right] + |L\rangle_B\left[|-5\rangle_B + |-4\rangle_B\right]\right) + |L\rangle_A\left(|R\rangle_B\left[|-5\rangle_B + |-4\rangle_B\right] + |L\rangle_B\left[|-10\rangle_B + |-9\rangle_B\right]\right). \tag{S5}$$

The extended topological transitions that arise from this heralded state are represented in Fig. S2**a**, with the spin textures illustrated at different points on the topological landscape. Notably, this choice of projection introduces more quasi-particles into the multiskyrmion structures, now forming a multiskyrmion at the poles. The multiskrymion structure emerging at the poles of the sphere can be tied to the choice of OAM charge for ℓ_{A_2}. (For example, if $\ell_{A_2} = -2$ instead, there would be two quasi particles in the multiskyrmion at the poles.) Additionally, a skyrmion number of $n_1 = -5$ is observed at the north pole of the sphere, before transitioning to $n_2 = -10$ for the equator states and $n_3 = -6$ at the south pole. Furthermore, by adjusting the input polarization to the q-plates to left-circular polarization, one can create skyrmions and multiskyrmions with positive skyrmion numbers, enabling distinct skyrmion textures to coexist within a single multiskyrmion. The spin texture of such a multiskyrmion with $n_2 = 10$

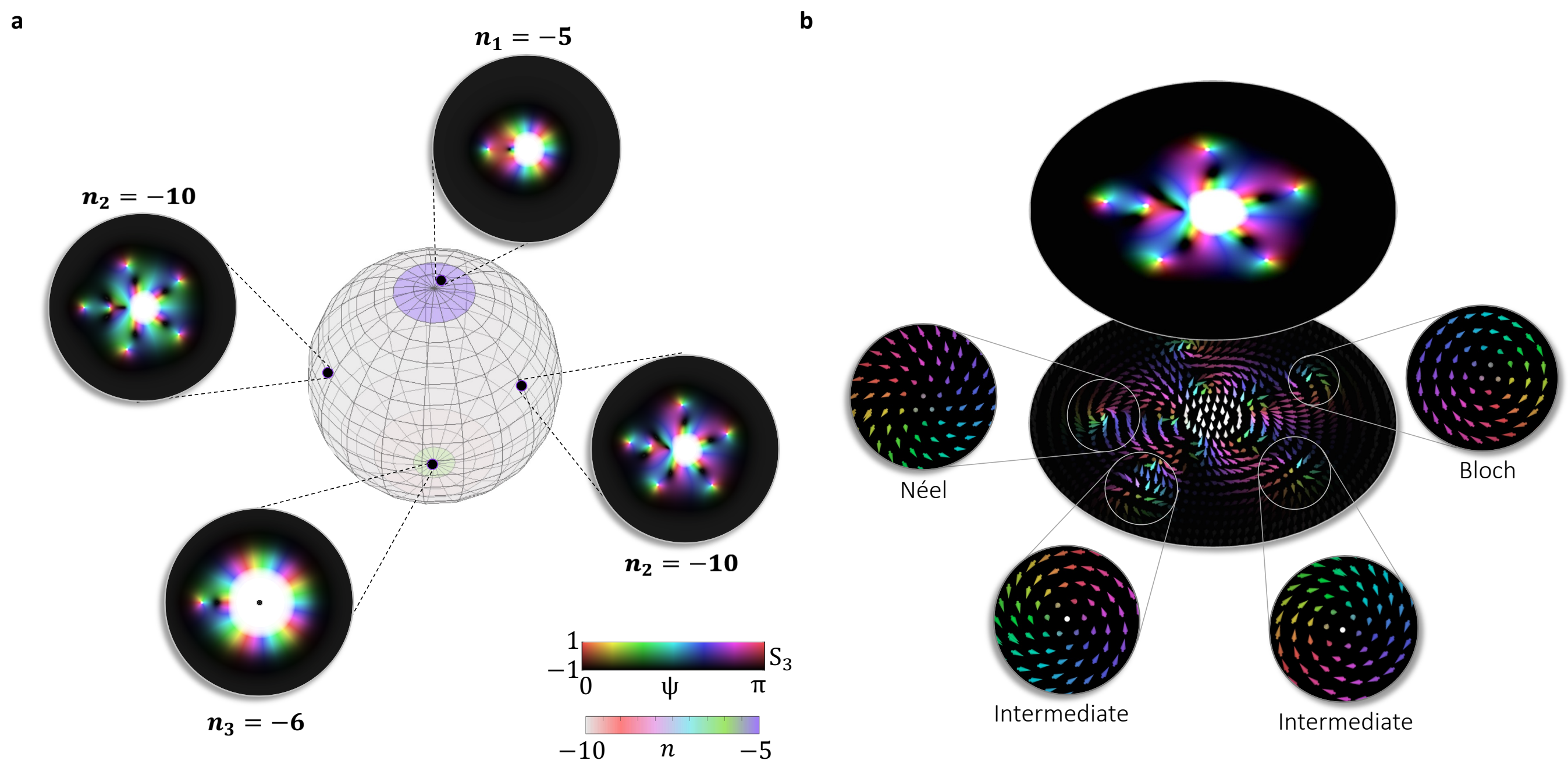

FIG. S2. **Extended topological transitions.** **a,** A switch between three different topological numbers for photon B is realised for a simulated case. The spin textures are illustrated at different points on the sphere for a state with **a,** $\ell_{A_1} = 0$ and $\ell_{A_2} = -1$, yielding a switching of the skyrmion number from $n_1 = -5$ at the north pole to $n_2 = -10$ at the equator and $n_3 = -6$ at the south pole. **b,** Simulated spin texture of a quantum multiskyrmion (with $n = 10$), with zoom-in insets revealing the embedded local polarization texture of the quasi-particles in the structure.

is visualised in Fig. S2, revealing the distinct local textures embedded within the broader field. Zooming into the individual localized skyrmions reveals distinct textures for each, ranging from hedgehog and spiral polarization textures characteristic of the Néel and Bloch-type skyrmions, respectively, to intermediate textures. It should be noted that, whilst our binary switch implementation only required polarization projections on the heralding photon, this extension requires both spatial and polarization projections, necessitating the use of an SLM on both arms.

EXTRACTION OF TOPOLOGICAL FEATURES

The topological features of our tripartite state, ρ, are derived from the correlations between photon B's internal spin and spatial DoFs after performing a projective measurement on photon A's spin DoF. Therefore, it is useful to map ρ onto the spatially-varying polarization density matrix given $\rho_{B|A}(\theta, \alpha, \vec{r}_B)$, which is given by

$$\rho_{B|A}(\theta, \alpha, \vec{r}_B) = {}_A\langle \theta, \alpha | \, \mathbb{1}_{B,\text{pol}} \, {}_B\langle \vec{r} | \; \rho \; |\theta, \alpha\rangle_A \, \mathbb{1}_{B,\text{pol}} \, |\vec{r}\rangle_B, \tag{S6}$$

where $|\theta, \alpha\rangle_A = \cos(\theta/2)|R\rangle_A + \sin(\theta/2)e^{i\alpha}|L\rangle_A$ is an arbitrary polarization measurement on photon A with $\theta \in [0, \pi], \alpha \in [0, 2\pi)$. In general, we can describe our tripartite state as

$$\rho = \sum_{ijklmn} c_{ijklmn} \, |P_i\rangle_A \, |P_j\rangle_B \, |\ell_k\rangle_B \; {}_A\langle P_l|_B\langle P_m| \; {}_B\langle \ell_n| \tag{S7}$$

where $|P_{i,j,l,m}\rangle \in \{|R\rangle, |L\rangle\}$, $k, n \in \{0, 1, 2, ..., d_{sp,B}\}$, and c_{ijklmn} are the density matrix coefficients. The corresponding spatially-varying density matrix is then given by

$$\rho_{B|A}(\theta, \alpha, \vec{r}_B) = \sum_{jkmn} A_{jkmn}(\theta, \alpha) \; \text{LG}^{p=0}_{\ell_k}(\vec{r}_B) \; \left(\text{LG}^{p=0}_{\ell_n}(\vec{r}_B)\right)^* \; |P_j\rangle_B \, {}_B\langle P_m| \tag{S8}$$

where $\text{LG}^{p=0}_{\ell}(\vec{r}_B)$ are the Laguerre-Gaussian functions with radial order, $p = 0$, and topological charge, ℓ, at $z = 0$ that results from projecting the OAM modes onto space, i.e., ${}_B\langle \vec{r} | \, \ell \rangle_B = \text{LG}^{p=0}_{\ell}(\vec{r}_B)$. $A_{jkmn}(\theta, \alpha)$ are the polarization

projection coefficients that are dependent on the projection on photon A, given by

$$A_{jkmn}(\theta,\alpha) = \sum_{jklm} \quad (c_{0jklm0}\cos^2(\theta/2) + c_{0jklm1}\cos(\theta/2)\sin(\theta/2)e^{-i\alpha} + c_{1jklm0}\cos(\theta/2)\sin(\theta/2)e^{i\alpha} + c_{1jklm1}\sin^2(\theta/2)). \tag{S9}$$

Next, we extract the spatially-varying quantum Stokes vector, $\vec{S}_{B|A}(\theta,\alpha,\vec{r}_B)$ whose components are given by $S_{B|A,p}(\theta,\alpha,\vec{r}_B) = \mathrm{Tr}\left(\sigma_p\, \rho_{B|A}(\theta,\alpha,\vec{r}_B)\right)$, where σ_p are the usual Pauli-spin matrices with $\sigma_0 = \mathbb{I}_2$. From this it is then straight-forward to verify that the quantum Stokes vector is given by

$$\vec{S}_{B|A}(\theta,\alpha,\vec{r}_B) = \begin{bmatrix} S_{B|A,0}(\theta,\alpha,\vec{r}_B) \\ S_{B|A,1}(\theta,\alpha,\vec{r}_B) \\ S_{B|A,2}(\theta,\alpha,\vec{r}_B) \\ S_{B|A,3}(\theta,\alpha,\vec{r}_B) \end{bmatrix} = \sum_{kn} \mathrm{LG}^{p=0}_{\ell_k}(\vec{r}_B)(\mathrm{LG}^{p=0}_{\ell_n}(\vec{r}_B))^* \begin{bmatrix} A_{0k0n}(\theta,\alpha) + A_{1k1n}(\theta,\alpha) \\ A_{0k1n}(\theta,\alpha) + A_{1k0n}(\theta,\alpha) \\ i\,(A_{0k1n}(\theta,\alpha) - A_{1k0n}(\theta,\alpha)) \\ A_{0k0n}(\theta,\alpha) - A_{1k1n}(\theta,\alpha) \end{bmatrix}. \tag{S10}$$

An example of a set of quantum Stokes parameters for the experimentally measured state with $\ell_{1,2,3} = 0,-3,-6$ for the projection with angles $\theta = 2.36$ rad, $\alpha =$1.57 rad is shown in Fig. S3e.

Finally, we can extract the topological structure of our tripartite state by substituting the quantum Stokes parameters into the usual skyrmion number integral,

$$n(\theta,\alpha) = \frac{1}{4\pi}\int_{\mathcal{R}^2} \vec{S}_{B|A}(\theta,\alpha,\vec{r}_B)\cdot\left(\frac{\partial \vec{S}_{B|A}(\theta,\alpha,\vec{r}_B)}{\partial x} \times \frac{\partial \vec{S}_{B|A}(\theta,\alpha,\vec{r}_B)}{\partial y}\right) dxdy. \tag{S11}$$

We note here that the skyrmion number is now parametrized by the projection angles of photon A, thereby yielding a new topological classification of these states, we term the "topological landscape" of the state. Within the main text, we observe two unique topological numbers per tripartite entangled state, suggesting that each state is characterized by a pair of topological numbers found by projecting photon A onto its basis states ($\theta = 0,\pi$) and any superposition state ($\theta \in (0,\pi), \alpha \in [0,2\pi]$).

GHZ STATE EXTRACTION AND TOPOLOGICAL DYNAMICS

In the main text we highlight an embedded GHZ-like state within our experimentally measured tripartite states and identify their characteristic Bell projection behaviour with tangible topological features and texture evolution allowing for the visualization of entanglement dynamics through topological dynamics.
To observe the embedded GHZ-like state, we expand Eq. 1 in the main text so that it reads

$$|\Psi\rangle = \quad |R\rangle_A\left(|R\rangle_B|\ell_1\rangle_B + |L\rangle_B|\ell_2\rangle_B\right) + |L\rangle_A\left(|R\rangle_B|\ell_2\rangle_B + |L\rangle_B|\ell_3\rangle_B\right) \tag{S12}$$

and, rearranging the terms, we find

$$|\Psi\rangle = \quad |R\rangle_A|R\rangle_B|\ell_1\rangle_B + |L\rangle_A|L\rangle_B|\ell_3\rangle_B + \left(|R\rangle_A|L\rangle_B + |L\rangle_A|R\rangle_B\right)|\ell_2\rangle_B. \tag{S13}$$

From this, the GHZ-like state appears as $|\psi^{GHZ}\rangle_{AB} = |R\rangle_A|R\rangle_B|\ell_1\rangle_B + |L\rangle_A|L\rangle_B|\ell_3\rangle_B$ which is interfered with a polarization entangled reference state, $|\psi^{Ref}\rangle_{AB} = (|R\rangle_A|L\rangle_B + |L\rangle_A|R\rangle_B)\,|\ell_2\rangle_B$. Given that the OAM Hilbert space of $|\psi^{GHZ}\rangle_{AB}$ is spanned by only two OAM states $\{|\ell_1\rangle, |\ell_3\rangle\}$, which excludes $|\ell_2\rangle$ that multiplies the reference state, we can extract the GHZ-like state by simply projecting photon B's OAM onto the two-dimensional subspace spanned by $|\ell_1\rangle$ and $|\ell_3\rangle$, $\hat{P} = |\ell_1\rangle\langle\ell_1| + |\ell_2\rangle\langle\ell_2|$.

As before, the topology of the state is identified post measurement of photon A's polarization. Projections onto the basis states of photon A for the GHZ state would collapse photon B into the separable states, $|R\rangle_B|\ell_1\rangle_B$ and $|R\rangle_B|\ell_3\rangle_B$, respectively; each possessing trivial topology, $n = 0$. Instead, projecting onto superposition states results in non-separable states with $|n| = |\ell_3 - \ell_1|$ (assuming $|\ell_3| \neq |\ell_1|$), with the topological feature. In the main text, an example of a GHZ-like state extracted from the tripartite state with OAM numbers $\ell_{1,2,3} = 0,-3,-6$ results in a topological landscape with $n = 0$ at the poles and $n = -6$ everywhere else.

We can then consider how the GHZ-like state is related to the initial tripartite state by studying the effect of the reference on the GHZ-like state. Firstly, the topological landscape is altered at the poles ($\theta = 0, \pi$) from $n = 0$ to $|n| = |\ell_1 - \ell_2|$, but is left unaltered everywhere else. This suggests that the state goes through a topological transition at the poles after adding the reference, but everywhere else the state is smoothly deformed. In the regime where the state undergoes a smooth deformation, the central skyrmionic quasi-particle is smoothly broken up into multiple quasi-particles with skyrmion numbers that sum to give the original one. Furthermore, when changing α in the superposition projection, the topological texture of the GHZ-like state is seen rotating; this is in contrast to the orbit and spin of the quasi-particle distribution seen once the reference is added. The nature of existent entanglement structures is thus emphasised through the observation of evolving topological structures within the state.

EXPERIMENTAL RESULTS

Experimental results for the example state with $\ell_1 = 0, \ell_2 = -3$ and $\ell_3 = -6$ (created using q-plates with $q = 1.5$) are shown in Fig. S3. A full QST was performed on the entangled photon state represented by the tomography data in Fig. S3**a** with zoomed in insets in **b**. The density matrix is then reconstructed as shown in Fig. S3**c**, with fidelity, $F = 0.95$ and purity $\gamma = 0.97$. Furthermore, to verify the non-local nature of this example state, we performed measurements to demonstrate a violation of the Clauser–Horne–Shimony–Holt (CHSH) Bell inequality. Joint projective measurements were carried out between photons A and B of our quantum state by selecting the polarisation settings $\{H, V, D, A\}$ on photon A and rotating the angle (θ_B) of the spatial analyser $|\theta_B\rangle = |0\rangle + e^{-i\theta_B}|-3\rangle$ for each setting in the $|R\rangle$ subspace of photon B as well as $|\theta_B\rangle = |-3\rangle + e^{-i\theta_B}|-6\rangle$ in the $|L\rangle$ subspace. Our results demonstrate a violation of the Bell inequality with a Bell parameter of $S = 2.43$ for the heralded state $|R\rangle_A |0\rangle_B + |L\rangle_A |-3\rangle_B$ and $S = 2.42$ for state $|R\rangle_A |-3\rangle_B + |L\rangle_A |-6\rangle_B$, with Bell curves shown in Fig. S3**e**.

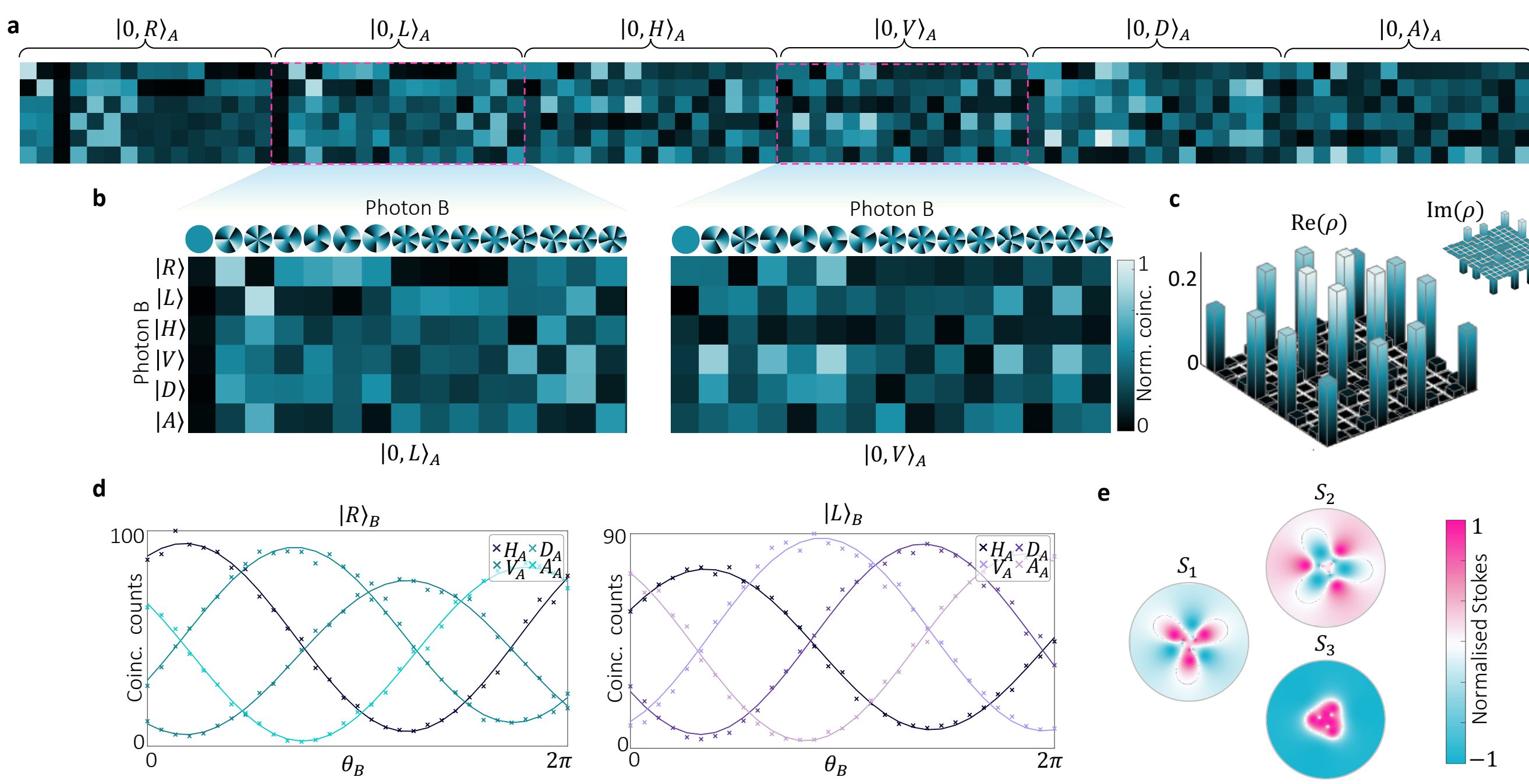

FIG. S3. **Experimental characterisation results. a,** The measurement matrix for the QST performed on the example state with $\ell_1 = 0, \ell_2 = -3$ and $\ell_3 = -6$, constructed by 6 polarization measurements on photon A, and 15 spatial measurements coupled with 6 polarization measurements on photon B, shown in the zoomed-in insets in **b**. **c,** Corresponding experimentally reconstructed density matrix extracted from the full QST with real and imaginary components. **d,** Violation of the Bell inequality. **e,** Stokes measurements retrieved directly from the tomography data.
